# Multivariate analysis reveals that BVDV field isolates do not show a close VN-based antigenic relationship to US vaccine strains

**DOI:** 10.1186/s13104-023-06410-2

**Published:** 2023-06-26

**Authors:** Ana Cristina S. Mosena, Hao Ma, Eduardo Casas, Rohana P. Dassanayake, Cláudio W. Canal, John D. Neill, Shollie M. Falkenberg

**Affiliations:** 1 Ruminant Disease and Immunology Research Unit, National Animal Disease Center, USDA, Agricultural Research Service, Ames, IA USA; 2 Laboratório de Virologia Veterinária, Faculdade de Veterinária, Universidade Federal do Rio Grande do Sul (UFRGS), Porto Alegre, Brazil; 3 College of Veterinary Medicine, Department of Pathobiology, Auburn University, Auburn, AL USA

**Keywords:** PCA, Antigenicity, pestivirus, BVDV, Virus neutralization

## Abstract

**Objective:**

Evaluate bovine viral diarrhea virus (BVDV) antigenicity by using virus neutralization titers (VNT) analyzed using the principal component analysis (PCA) from antisera generated against US-based vaccine strains against both US-origin field isolates and non-US-origin field isolates.

**Results:**

Data from both independent analyses demonstrated that several US-origin and non-US-origin BVDV field isolates appear to be antigenically divergent from the US-based vaccine strains. Results from the combined analysis provided greater insight into the antigenic diversity observed among BVDV isolates. Data from this study further support genetic assignment into BVDV subgenotypes, as well as strains within subgenotypes is not representative of antigenic relatedness. PCA highlights isolates that are antigenically divergent from members of the same species and subgenotype and conversely isolates that belong to different subgenotypes have similar antigenic characteristics when using antisera from US-based vaccine isolates.

**Supplementary Information:**

The online version contains supplementary material available at 10.1186/s13104-023-06410-2.

## Introduction

Three ruminant pestiviruses, commonly referred to as Bovine Viral Diarrhea Virus (BVDV) 1, BVDV-2, and HoBi-like pestivirus (HoBiPeV), additionally characterized as Pestivirus A, B, and H, can cause respiratory and intestinal disease in cattle that lead to decreased production [1, 2]. BVDV species have broad genetic diversity and are currently classified into 21 subgenotypes for BVDV-1 (BVDV-1a to BVDV-1u) and 4 subgenotypes for BVDV-2 (BVDV-2a to BVDV-d), based on genome phylogeny [3]. The antigenicity, as measured by virus neutralizing titer (VNT), of this broad genetic diversity within isolates from the same species is still not well understood [4–7]. Additionally, it is unknown what and where the genetic changes occur that result in antigenic differences between genetically similar isolates.

Studies previously utilizing the principal component analysis (PCA) used polyclonal antisera generated by exposing cattle to six different typical US-based BVDV vaccine strains (3 BVDV-1a and 3 BVDV-2a strains, all isolated in North America) against US-origin [8] or non-US-origin [9] field isolates, and against the vaccine strains used to generate the antisera. Results from these previous studies demonstrated that, using VNT data and PCA statistical approach for data visualization, isolates from the same subgenotype do not always cluster into antigenically similar groups. Some isolates are divergent and have no VNT pattern similar to any of the other isolates using PCA. In addition, strains currently used in available BVDV vaccines appear to be antigenically divergent from numerous field isolates [8, 9].

Since only a few isolates from each subgenotype were analyzed in the previous studies and studies were evaluated based on geography [8, 9], it is possible to only suggest antigenic groups and determine if antigenic patterns exist that could be explained by genomic features. Therefore, combining the two datasets to include more isolates from different subgenotypes and geographic origins allow for moderate robust analysis to evaluate VN-based antigenic relationships between field isolates and strains used in vaccines.

## Materials and methods

Field isolates representing BVDV-1 (1a to 1i and 1k) and 2 (2a, 2b, and 2c) subgenotypes, along with one HoBiPeV isolate, previously analyzed independently [8, 9] were combined for the current analysis. The two previous analyses represented isolates from distinct geographical locations and BVDV subgenotype diversity (US-origin and non-US-origin) [8, 9]. The combination of the two independent analyses included 31 cp. and ncp BVDV-1 field isolates from Italy (1e, 1f, 1 g and 1k subgenotypes), Swiss (1e, 1 h and 1k), United Kingdom (1a, 1d, 1e and 1i) and USA (1a, 1b, 1c and 1i), and six USA BVDV-2 field isolates (2a, 2b and 2c subgenotypes). One Brazilian HoBiPeV isolate was included in the study. Additionally, seven cp. US-origin strains currently used in modified-live vaccine formulation were also included (C24V_1a, Singer_1a and NADL_1a and 125_2a, 296_2a, 53637_2a and 5912_2a).

From the seven selected vaccine strains, specific antiserum was generated against six strains (C24V_1a, Singer_1a, NADL_1a, 125_2a, 296_2a, and 53637_2a) and used in VN assay against the field isolates to obtain VNT. Details regarding antisera generation and approval of study procedures reviewed and approved by the Institutional Animal Care and Use Committee at the National Animal Disease Center (protocol #ARS-2017–673) have previously been reported [8–10].

Details regarding complete genome sequencing, detailed phylogeny, and BVDV isolate characterization are previously described in literature, in addition to GenBank accession numbers [8, 9]. Cell maintenance, virus propagation, VNT procedure and determination, and description of PCA as a tool for BVDV antigenic characterization are previously described [8, 9].

## Results

**Fig. 1:**
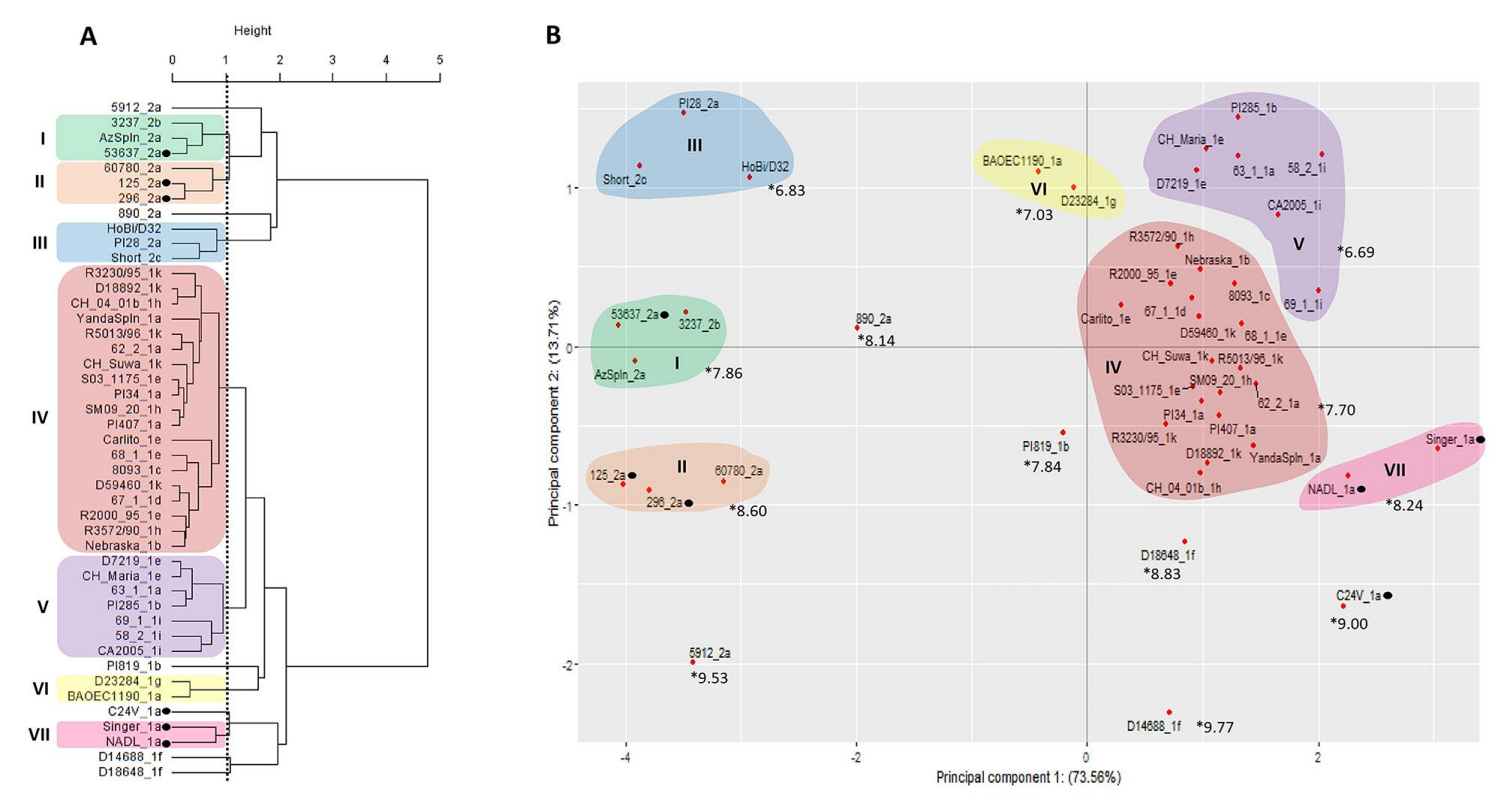
Methods to evaluate similar antigenic clustering using 45 BVDV strains (9 BVDV-1a, 3 BVDV-1b, 1 BVDV-1c, 1 BVDV-1d, 6 BVDV-1e, 2 BVDV-1f, 1 BVDV-1 g, 3 BVDV-1 h, 3 BVDV-1i, 5 BVDV-1k, 7 BVDV-2a, 1 BVDV-2b, 1 BVDV-2c, and 1 HoBi) against antisera generated against three BVDV-1a vaccine strains (C24V, Singer, and NADL) and three BVDV-2a strains (53637c, 125c, and 296c). Vaccine strains are highlighted with symbols. (**A**) Cluster analysis dendrogram using Ward’s method using the variation from both principal components 1 and 2 to cluster strains into like groups. (**B**) Principal component scatter plot displaying independent contribution of the first two principal components accounting for the largest variation in the samples. The average neutralizing titer from a cluster against the antisera is marked with *

**Fig. 2:**
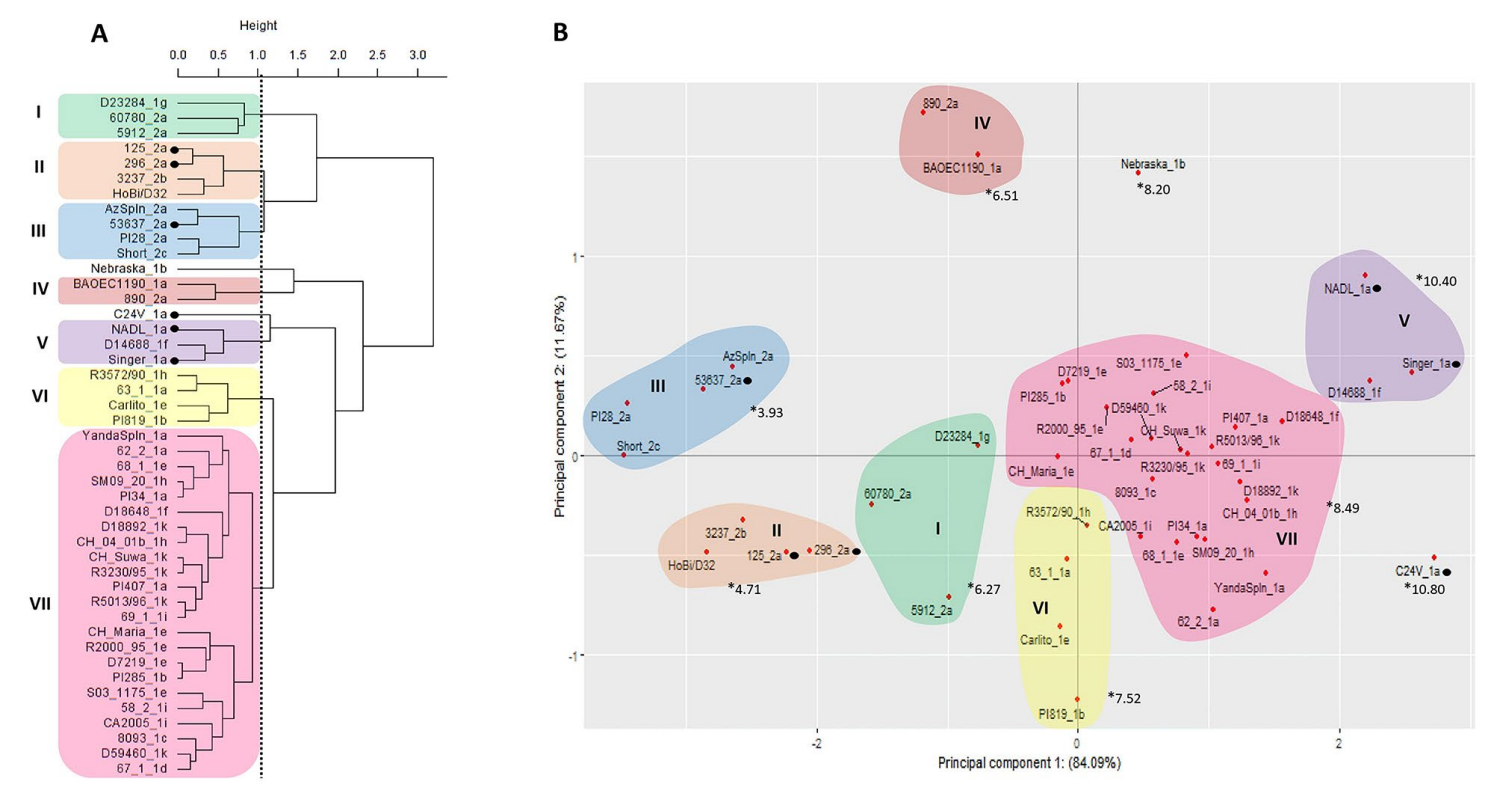
Methods to evaluate similar antigenic clustering using 45 BVDV strains (9 BVDV-1a, 3 BVDV-1b, 1 BVDV-1c, 1 BVDV-1d, 6 BVDV-1e, 2 BVDV-1f, 1 BVDV-1 g, 3 BVDV-1 h, 3 BVDV-1i, 5 BVDV-1k, 7 BVDV-2a, 1 BVDV-2b, 1 BVDV-2c, and 1 HoBi) and antisera generated against three BVDV-1a vaccine strains (C24V, NADL, and Singer). Vaccine strains are highlighted with symbols. (**A**) Cluster analysis dendrogram using Ward’s method using the variation from both principal components 1 and 2 to cluster strains into like groups. (**B**) Principal component scatter plot displaying independent contribution of the first two principal components accounting for the largest variation in the samples. The average neutralizing titer from a cluster against the antisera is marked with *

**Fig. 3:**
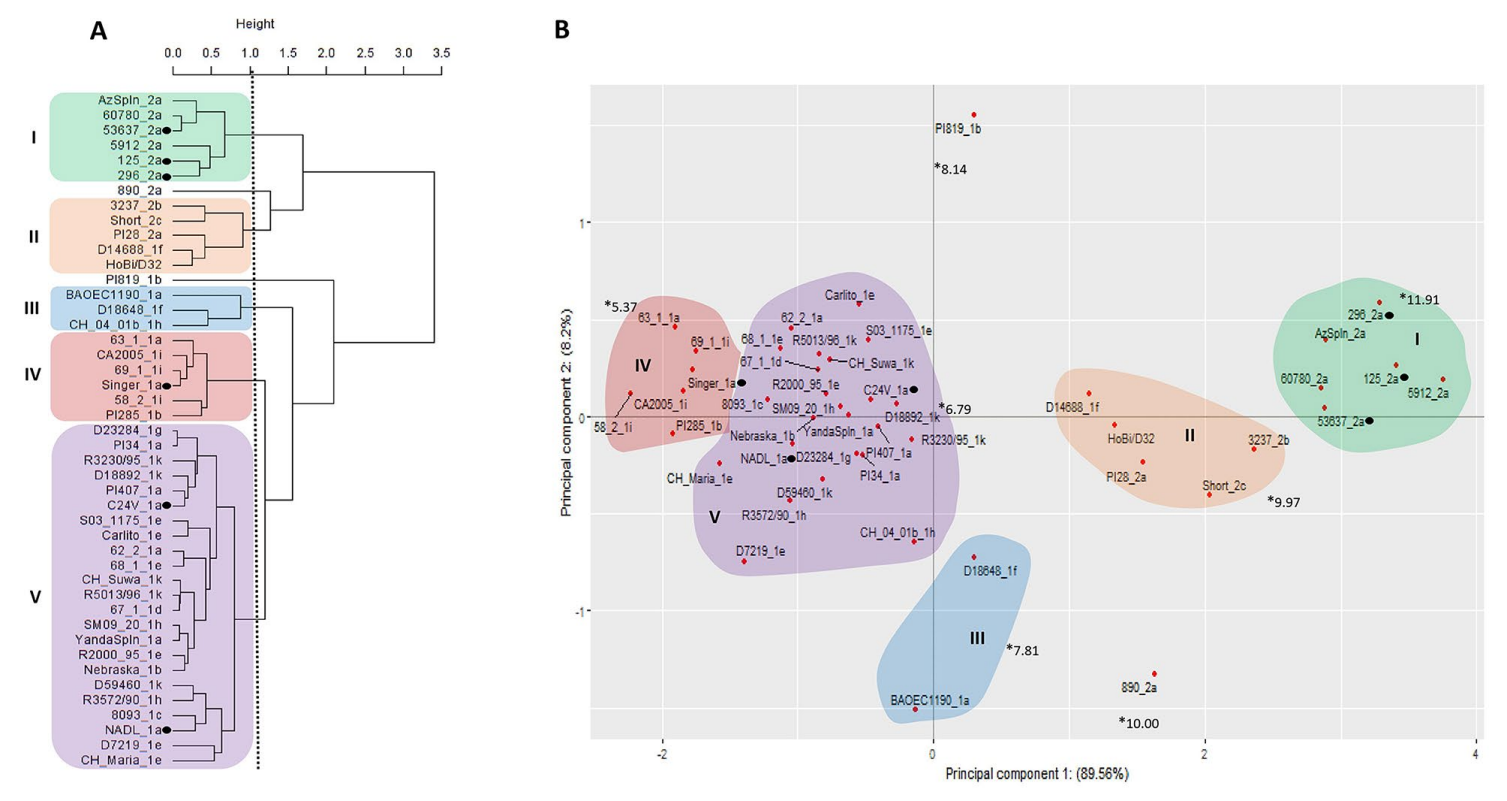
Methods to evaluate similar antigenic clustering using 45 BVDV strains (9 BVDV-1a, 3 BVDV-1b, 1 BVDV-1c, 1 BVDV-1d, 6 BVDV-1e, 2 BVDV-1f, 1 BVDV-1 g, 3 BVDV-1 h, 3 BVDV-1i, 5 BVDV-1k, 7 BVDV-2a, 1 BVDV-2b, 1 BVDV-2c, and 1 HoBi) and three BVDV-2a antisera generated against vaccine strains (296c 53637c, and 125c). Vaccine strains are highlighted with symbols. (**A**) Cluster analysis dendrogram using Ward’s method using the variation from both principal components 1 and 2 to cluster strains into like groups. (**B**) Principal component scatter plot displaying independent contribution of the first two principal components accounting for the largest variation in the samples. The average neutralizing titer from a cluster against the antisera is marked with *

Non-US-origin and US-origin datasets [8, 9] were combined and analyzed using both BVDV-1a and BVDV-2a antisera titers (Fig. 1), only BVDV-1a antisera titers (Fig. 2) and only BVDV-2a antisera titers (Fig. 3). A height of 1 within the PCA cluster dendrogram was used as the criteria to characterize strains and isolates into like groups (Figs. 1A and 2 A, and 3 A). The PCA two-dimensional approach generated a scatter plot where the contribution of each principal component (PC) value can be visualized in the graphic (PC1 represented in the X axis and PC2 in the Y axis). When viruses clustered into like groups in the PCA dendrogram, those were denoted within the PCA scatter plot.

### All antisera (BVDV-1 and BVDV-2)

The combined non-US/US-origin dataset was evaluated for antigenic relatedness using VNT against the six vaccine antisera. PCA revealed seven clusters composed of multiple isolates (Fig. 1). The BVDV-1 branch was composed of four clusters, with BVDV-1 vaccines strains Singer and NADL forming one cluster (VII, Fig. 1), while the vaccine strain C24V formed an individual branch. No field isolates clustered with vaccine strains. Isolates D23284_1g D14688_1f and PI819_1b isolates formed individual branches (Fig. 1A). All the US-origin and non-US-origin 1i isolates (CA2005, 58_2 and 69_1) were contained in cluster V, along with other isolates from 1a, 1b and 1e subgenotypes (Fig. 1). The cluster IV was composed by the majority of the isolates representing several subgenotypes (1a, 1b, 1c, 1d, 1e, 1k and 1 h) from US-origin and non-US-origin. In the BVDV-2 branch, three clusters were formed by more than one virus (Fig. 1 Two BVDV-2a isolates (vaccine strain 5912_2a and isolate 890_2a, Fig. 1) formed individual branches.

The PC1 represented by the X axis in the PCA scatter plot was representative of BVDV species differences, accounting for approximately 73% of the variability (Fig. 1B). Isolates BAOEC1190_1a, D23284_1g, and PI819_ 1b formed individual branches within the PCA dendrogram within the BVDV-1 branches. Although in the PCA scatter plot these isolates were oriented on the opposite side of the Y axis from other BVDV-1 isolates, and were oriented on the same side on the Y axis associated with the BVDV-2 isolates.

To determine if the neutralizing antibody titers could differ between clusters, average VNTs were compared among cluster groups and individual isolates. The clusters’ average results (Complementary Table) were identified in Fig. 1B (denoted by *). The highest titers were associated with individual vaccine strains and clusters, which is expected, but the VNT for isolate D14688_1f had the highest titer of 9.77. The lowest VNTs were associated with clusters III and V. Interestingly BVDV-2 isolate PI28_2a is within cluster III whereas all other isolates in these clusters belong to other subgenotypes not contained in current BVDV vaccines.

### BVDV-1 antisera

When using BVDV-1 antisera (C24V_1a, NADL_1a, and Singer_1a antisera; Fig. 2) there were differences in clustering. PCA revealed seven clusters with the BVDV-1 branch composed of four clusters, and only the Nebraska_1b isolate and the C24V_1a vaccine strain formed an individual branch (Fig. 2A). Additionally, the 890_2a isolate was divergent and clustered with isolate BAOEC1190_1a within the BVDV-1 main branch. In the BVDV-2 branch, three clusters were formed by more than one virus and no individual branches.

The PC1 represented by the X axis in the PCA scatter plot was again representative of BVDV species differences, accounting for approximately 84% of the variability (Fig. 2B). Spatially within the PCA scatter plot multiple BVDV-1 isolates in clusters I, IV, VI, and VII while initially clustering within the BVDV-1 branch of the dendrogram, were oriented on the opposite side of the Y axis from other BVDV-1 isolates and instead oriented on the same side on the Y axis associated with the BVDV-2 isolates (Fig. 2B).

Average VNTs were compared among cluster groups and individual isolates. The clusters’ average results (Complementary Table) were identified in Fig. 2B (denoted by *). The highest titers were associated with individual BVDV-1 vaccine strains and clusters, which is expected. The lowest VNT were associated with clusters containing BVDV-2 isolates, although BVDV-1 isolates D23284_1g and BAOEC1190_1a were also contained in these clusters (I and IV).

### BVDV-2 antisera

When using BVDV-2 antisera (125_2a, 296_2a, and 53637_2a; Fig. 3), there were differences in clustering. PCA revealed five clusters with the BVDV-1 branch composed of three clusters, and only the PI819_1b isolate forming an individual branch (Fig. 3A). In the BVDV-2 branch, two clusters were formed by more than one virus and only the 890_2b isolate formed an individual branch (Fig. 3A). Additionally, the D14688_1f isolate showed a divergent behavior and was contained in cluster II with BVDV-2a, 2b, 2c, and HoBiPeV isolates within the BVDV-2 main branch.

The PC1 represented by the X axis in the PCA scatter plot was again representative of BVDV species differences, accounting for approximately 90% of the variability (Fig. 3B). Spatially within the PCA scatter plot BVDV-1 isolate (D18648_1f) in cluster III and PI819_1b that formed an individual branch, while initially within the BVDV-1 branch of the dendrogram, were oriented on the opposite side of the Y axis from other BVDV-1 isolates and oriented on the same side on the Y axis associated with the BVDV-2 isolates (Fig. 3B).

Average VNTs were compared among cluster groups and individual isolates. The clusters’ average results (Complementary Table) were identified in Fig. 3B (denoted by *). The highest titers were associated with BVDV-2 vaccine strain clusters, and the lowest VNTs were associated with clusters containing BVDV-1 isolates, which is expected. Although, BVDV-1 isolate D14688_1f was contained in cluster II which had a high average titer of 9.97.

## Discussion

This study is the sequence and complement of previous studies [8, 9], to compare and understand how the PCA methodology behaves when more field isolates are added to the dataset. As in previous studies, there was no correlation between genetic and antigenic clusters. However, as corroborated previously, patterns for individual isolates were observed, especially for isolates that are dissimilar from other field isolates. The factors that contribute to antigenic similarity (clusters) or divergence were not analyzed in this study, although the clusters’ average VNTs were included in this analysis to further expand the understanding of VNT relationships. The range of VNTs that compose the clusters’ averages did differ between clusters and isolates and strains with the lowest average titers clustered together (Complementary Table). Additionally, using only BVDV species-specific antisera (BVDV-1 or BVDV-2) helped provide further indication of antigenic dissimilar isolates. This is most evident for isolate BAOEC1190_1a which consistently had lower VNT regardless of antisera used for comparison (Figs. 1B and 2B, and 3B), whereas isolate D14688_1f consistently had higher VNT regardless of the BVDV species-specific antisera (Figs. 1B and 2B, and 3B, Complementary Table). While not as evident, another BVDV-1f isolate (D18648_1f) had similar observable trends (Fig. 1B, Complementary Table) as observed by having BVDV-2 antisera titers higher than other BVDV-1 isolates (Fig. 3B).

Additionally, PI819_1b had a VNT of 7.84 when using all antisera but formed an individual branch and did not cluster with the majority of BVDV-1 isolates that had a similar average VNT of 7.7 (Fig. 1B, Complementary Table). Although, when only using BVDV-1 antisera, the PI_819_1b clustered with other BVDV-1 dissimilar isolates that had an average VNT of 7.52 (Fig. 2B). Whereas when using BVDV-2 antisera PI_819 formed an individual branch and had a VNT of 8.14 (Fig. 3B). Those observations could suggest that the BVDV-2 antisera neutralized a BVDV-1 isolate more effectively than the BVDV-1 antisera. Isolates that appear to be more broadly neutralized warrant further investigation in the future as potential vaccine candidates. Based on this collective and combined study, more isolates from more subgenotypes will provide further information about the breadth of antigenic relationships with US-origin vaccine antisera, thereby strengthening the methodology to determine relationships or criteria for clustering of viruses. With a wider range of data on subgenotypes variability, the PCA could identify dissimilar and antigenically divergent isolates. Those isolates could be used for the identification of genetic characteristics that influence the antigenicity of isolates, improving, for example, the production of more efficient and broader protection pestivirus vaccines.

### Limitations

- Due to laborious VN test execution and limitation of isolated strains in the laboratory virus library, some subgenotypes were represented by a few field isolates, and not common or geographically specific subgenotypes were not included in this study.
- In this study only neutralizing antibody titers were studied. Pestiviruses antigenic response in hosts is composed of multiple factors, being serum antibodies only one of them. The cellular immune response is an important factor in antigenic response, but it was not included in this study.

## Electronic supplementary material

Below is the link to the electronic supplementary material.

Supplementary Material 1

## Data Availability

All data used in this study are available and were obtained from previous publications:—Mosena, A. C. S., Falkenberg, S. M., Ma, H., Casas, E., Dassanayake, R. P., Walz, P. H., … & Neill, J. D. (2020). Multivariate analysis as a method to evaluate antigenic relationships between BVDV vaccine and field strains. Vaccine, 38(36), 5764–5772. Doi: 10.1016/j.vaccine.2020.07.010, A. C. S., Falkenberg, S. M., Ma, H., Casas, E., Dassanayake, R. P., Booth, R., … & Neill, J. D. (2022). Use of multivariate analysis to evaluate antigenic relationships between US BVDV vaccine strains and non-US genetically divergent isolates. Journal of virological methods, 299, 114,328. Doi: 10.1016/j.jviromet.2021.114328.
